# Impact of epilepsy and its treatment on brain metastasis from solid tumors: A retrospective study

**DOI:** 10.3389/fneur.2022.967946

**Published:** 2022-10-21

**Authors:** Marta Maschio, Andrea Maialetti, Diana Giannarelli, Tatiana Koudriavtseva, Edvina Galiè, Alessandra Fabi

**Affiliations:** ^1^Center for Tumour-Related Epilepsy—Neuro-oncology Unit, IRCCS Regina Elena National Cancer Institute, Rome, Italy; ^2^Clinical Trial Design and Analysis Scientific Directorate, Department of Woman and Child Health and Public Health, Fondazione Policlinico Universitario A. Gemelli IRCCS, Rome, Italy; ^3^Medical Direction, IRCCS Regina Elena National Cancer Institute, Rome, Italy; ^4^Neuro-oncology Unit, IRCCS Regina Elena National Cancer Institute, Rome, Italy; ^5^Unit of Precision Medicine in Breast Cancer, Scientific Directorate, Department of Woman and Child Health and Public Health, Fondazione Policlinico Universitario A. Gemelli IRCCS, Rome, Italy

**Keywords:** brain metastasis, seizures, epilepsy, ASMs, interaction, side effects, chemotherapy

## Abstract

**Introduction:**

Retrospective observational study on medical records of patients with epilepsy related brain metastases (BM) to evaluate efficacy, safety and possible interaction with cancer treatment of different anti-seizure medications (ASMs) and the risk of seizures.

**Materials and methods:**

We consecutively reviewed all medical records of epilepsy-related BM patients from 2010 to 2020 who were followed for at least one month at the Brain Tumour-related Epilepsy Center of the IRCCS Regina Elena National Cancer Institute Rome, Italy.

**Results:**

We selected 111 cancer patients. Of these, only 42 had at least undergone a second neurological examination. In the whole population, 95 (85.2%) had seizures and 16 patients had no seizures (14.4%). The most frequently first ASM prescribed was LEV (40.5%). We observed a significant correlation between tumor site and probability of having seizures, but not between seizure type and age (>65 or <65 years). Among 42 patients, 26 were administered levetiracetam, followed by oxcarbazepine. Until the last follow-up, 19 never changed the first ASM, maintained the same dosage and remained seizure free. After a median of 7 months, 16 (38.1%) required changes in therapeutic treatment due to inefficacy. At the last follow-up, 24 patients (57.1%) were seizure free. Eighteen patients (42.8%) never achieved freedom from seizures despite had at least 2 therapy changes. Two patients changed ASM due to adverse events and 1 to phenobarbital owing to the interaction with cancer treatment. The mean daily dose of first ASM in all 42 patients was very close to the Defined Daily Dose (DDD).

**Conclusion:**

In BM patients seizure incidence could be underestimated; a team evaluation performed by oncologist and neurologist together, could guarantee an accurate taking care of both oncological illness and epilepsy, in this fragile patient population. More than 50% of our patients respond to monotherapy with new generation ASMs. Furthermore we deemed in patients receiving chemotherapy the choice of ASM should consider possible interactions with antitumor therapies, for this reason newer generation ASMs should be the preferred choice. It is necessary to get close to the DDD before considering an ASM ineffective in seizure control.

## Introduction

Adult patients with cancer could develop brain metastases (BM) in ~10–40% of cases, and the incidence of seizures in this population ranges between 15 and 25% (1, 2). They could be experienced as disease-related presenting symptoms or during the course of the disease itself (3, 4). There are very little data available on the impact of epilepsy in BM patients in the literature, but the burden of seizures on the quality of life in these patients is enormously vast (5, 6). As for brain tumor patients, epilepsy is considered the most important risk factor for long-term disability (3), in particular postoperative seizures which are associated with considerable morbidity, longer length of hospital stay, and higher rates of readmission (7). Furthermore, concerns about seizure-associated morbidity often prompt physicians to seek to aggressively treat or prevent epilepsy; despite this, there is a dearth of studies to guide them in selecting the most appropriate antiseizure medication (ASMs) in this patient population (8). Seizures can be effectively treated or prevented with a number of ASMs, but these agents have associated risks and adverse events (AEs).

It is for this reason the decision to treat a patient with ASM must be made carefully, balancing the efficacy in controlling seizures with the occurrence of potential AEs or possible interactions with systemic therapies (8). In the last few decades, many advances have been made in cancer treatment concerning the use of biological, immunological molecules other than new chemotherapeutic agents. The clinical benefit of the new oncological treatments has a great impact on disease outcome. Some new anticancer drugs, biological molecules (for example, anti-Alk in lung cancer, anti-HER2 molecules for advanced breast cancer) and immunological (checkpoint inhibitors, anti-PD/PDL1) drugs, have shown an evident activity on brain metastases with the ability to control neurological symptoms (9–13).

Current data in the literature regarding patients with brain tumors indicate that new generations of ASMs are the best choice to reduce the risk of possible AEs and drug interactions (8). To date, there have been only a few studies conducted on the efficacy and tolerability of new generations of ASMs during systemic therapy in BM patients (5–8).

For these reasons, we decided to undertake a retrospective observational study on patients with epilepsy-related BM. The overall aim of the study was to evaluate the efficacy and safety of different ASMs and the risk of seizures.

## Materials and methods

Retrospective monocentric study. We consecutively reviewed medical records of all BM patients who acceded for a first visit to Brain Tumour-related Epilepsy Center of the IRCCS Regina Elena National Cancer Institute Rome Italy from 2010 to 2020 and for whom the following information were available:

- Diagnosis of BM made by neuroimaging/biopsy/surgery.
- Demographic variables: sex, age (>18 and < 80 years), education.
- Date of diagnosis, histological type, and site of the primary tumor.
- Date of diagnosis, histological type, and site of BM.
- Date and type of neurosurgery (gross total resection, partial resection, biopsy, no intervention).
- Date and type of chemotherapy/radiotherapy for primary tumor and BM.

Only for patients who experienced one or more seizure related to BM and who had at least a second neurological visit, the following information were collected:

- Date and type of the first seizure (according to ILAE-International League Against Epilepsy classification: focal aware, focal with impaired awareness, focal to bilateral tonic-clonic, and generalized seizures) (14);
- Date, type, and mean daily dose of the first and subsequent ASMs (carbamazepine-CBZ, clobazam-CLZ, phenytoin-PHT, phenobarbital-PB, valproic acid-VPA, Tiagabine-TGB, lamotrigine-LTG, levetiracetam-LEV, oxcarbazepine-OXC, pregabalin-PGB, topiramate-TPM, zonisamide-ZNS, Lacosamide-LCM, and Perampanel-PER);
- ASMs regimes: monotherapy or polytherapy;
- Eventual ASMs change or dosage modifications and reason for change (inefficacy, AEs);
- AE occurrence during ASM therapy; and
- Date of the last follow-up available.

With reference to the onset of seizures after diagnosis, we did not use a specific cut-off rate as we could not exclude the occurrence of seizures even after prolonged periods of time.

Patients were excluded if their medical records reported: ages <18 and> 80; patients with a long history of seizures preceding metastasis diagnosis and judged by the caring physician to be unrelated to brain metastases; Karnofsky Performance Status (KPS) <50 points (15).

The period for changing the first ASM due to lack of efficacy and/or toxicity was considered a specific endpoint. The diagnosis of epilepsy, the classification of seizures, and the choice of antiepileptic therapy were made in accordance with ILAE guidelines (14). Patients were considered seizure free if they experienced no seizures until the last follow-up available on unchanged first ASM treatment. The ASM mean daily dose for each patient was calculated with respect to the defined daily dose (DDD), which is the assumed average maintenance dose per day for a drug used for its main indication in adults, as indicated by the World Health Organization (WHO) (16).

An “adverse event” was defined as any unfavorable and unintended sign, symptom, or disease temporally associated with the medical treatment (17). An AE may or may not be related to the medical treatment. Symptoms related to tumor progression were not considered to be an AE. AEs were categorized as sedation, mood disorder/irritability, vertigo, gastrointestinal, hematological, and rash. All AEs were recorded in our database. An AE was attributed to a specific ASM if the attending physician had evaluated that the AE in the medical record was directly related to the drug or if the AE only occurred or aggravated after starting or increasing the dose of a specific ASM. We defined an AE as intolerable if it led to a decrease in dose or cessation of an ASM.

The information was collected through a formatted Excel worksheet. Control of the quality and completeness of collected data was performed before analysis. In order to reduce the selection bias, all the medical records were examined consecutively and all consecutive patients who met the selection criteria were collected. This study was approved by the Ethics Committee (RS 1498/21).

### Endpoints

Primary endpoint was to evaluate the efficacy of different antiepileptic therapies on seizure control in patients with BM-related epilepsy.

Secondary endpoints were to detect the incidence of ASMs-related AE; evaluate whether the efficacy of ASMs is modified by concomitant presence/absence of systemic antineoplastic therapy; to detect whether a seizure risk correlates to any oncological variable; to evaluate whether efficacy of a different ASM is correlated to the mean daily dose, the possible association between oncological variables and the appearance of seizures.

### Efficacy variables

Primary efficacy variable was seizure freedom (18–20) and the retention time (therapeutic failure: time until the first ASM switched to another ASM or add a second ASM due to lack of efficacy and/or toxicity).

Secondary efficacy variables were: time of appearance of AEs compared to the time of introduction of the ASM; correlation between time to the appearance of the second and last seizure in patients with the same ASM underwent different systemic therapies; correlation between the occurrence of seizure and any oncological variable; correlation between mean daily dose of different ASMs and DDD in patient seizure free and non-seizure free.

### Statistical analysis

Data analysis was mainly descriptive and was performed for the entire series of patients and the subset evaluable for follow-up. Qualitative variables were summarized with absolute frequencies and percentages, while the mean and standard deviations (SD), medians and interquartile range (IQR) were used for quantitative items as appropriate. Quantitative variables, when needed, were dichotomized using the median value as a cut-off. Time-to-event analysis (e.g., time to modify first ASM for inefficacy and/or toxicity) was performed with the Kaplan-Meier method, and differences were evaluated with the log-rank test. Independent predictors of time to treatment change were assessed with Cox proportional hazard models. The risks were expressed as Hazard Ratios (HR) with 95% Confidence Intervals (95% CI). Different models were used according to age (continuous variable; categorical variable, median age as cut-off value) and drugs (active principle; enzyme inducers vs. non-inducers). Missing values were reported for each item, and no substitutions were made. As this was an exploratory 174 study, a calculation of the sample size was not planned. Data were analyzed using the statistical package IMB SPSS Statistics v.21.0.

## Results

Among all the patients treated in the center between 2010 and 2020, we selected 111 consecutive patients who met the selection criteria. Among these patients, 69 were lost either because they came from other regions or because of poor compliance. Therefore, it was possible to carry out at least a second neurological examination only in 42 patients, who experienced one or more seizure during follow-up.

Results on 111 patients (see Table 1 for details).

**Table 1:** Demographic and oncological variables in the whole population (*n* = 111 patients) at the first neurological examination.

**Patient characteristics**	***N*. (%)**
**Gender**	
Male	54 (48.6%)
Female	57 (51.4%)
AGE (mean, SD) (median, IQR)	54 (±13) (54, 45–65)
**Histology**	
Lung (NSCLC, SCLC)	48 (43.2%)
Breast	29 (26.1%)
Melanoma	18 (16.2%)
Colon	5 (4.5%)
Bladder	4 (3.6%)
Other	7 (6.3%)
**Brain metastases site**	
Frontal	24 (21.6%)
Temporal	6 (5.4%)
Parietal	16 (14.4%)
Occipital	6 (5.4%)
Multilobular	57 (51.4%)
Cerebellar	2 (1.8%)
**Side of brain metastases**	
Left	22 (19.8%)
Right	31 (27.9%)
Both	17 (15.3%)
Unknown	41 (36.9%)

SD, standard deviation; IQR, interquartile range; NSCLC, non-small cell lung cancer; SCLC, small cell lung cancer.

54 males and 57 females (51.4%), mean age at the first visit in the epilepsy center was 54 (±13 SD) years and the median was 54, IQR = 45–65. The most frequent cancers were lung cancer (48 patients, 43.2%) and breast cancer (26.1 patients, 26.1%). The most frequent sites of brain metastases were: multilobular (57 patients, 51.4%), frontal lobe (24 patients, 21.6%) and parietal lobe (16 patients, 14.4%). In 31 patients, the metastases were located on the right side (31 patients, 27.9%).

Among the 111 patients, 16 have not seizures (14.4%) and 95 (85.2%) have seizures. Fifty-five have focal aware seizures (50.5%), 14 focal with impaired awareness (12.6%), 19 focal to bilateral tonic clonic seizures (17.1%), 2 (1.8%) with both focal aware and focal with impaired awareness seizures and 4 (apparently) generalized (3.6%).

The most frequent first ASM prescribed was LEV (45 patients, 40.5%), followed by OXC (25 patients, 22.5%). Regarding the probability of having seizures, we found no statistically significant correlation between seizure occurrence and gender (male vs. female, *p* = 0.91) and primary cancer (lung vs. breast vs. melanoma vs. other, *p* = 0.62). The brain site significantly affects the probability of having seizures (all patients with parietal metastases have seizures; *p* = 0.012).

We observed no significant correlation between seizure type (focal aware, focal with impaired awareness, and generalized seizures) and gender (*p* = 0.75), BM site (*p* = 0.98), primary cancer (*p* = 0.56), and age (<65 or >65 years) (*p* = 0.07) (21).

Results on 42 patients (see Table 2 for details).

**Table 2:** Demographic and oncological variables of patients who underwent at least a second neurological examination (*n* = 42 patients).

**Patient characteristics**	***N*. (%)**
**Gender**	
Male	18 (42.9%)
Female	24 (57.1%)
AGE (mean, SD) (median, IQR)	52 (±12) (52, 45–62)
**Histology**	
Lung (NSCLC, SCLC)	21 (50%)
Breast	13 (31%)
Melanoma	4 (9.5%)
Bladder	2 (4.8%)
Other	2 (4.8%)
**Brain metastases site**	
Frontal	9 (21.4%)
Temporal	4 (9.5%)
Parietal	7 (16.7%)
Occipital	4 (9.5%)
Multilobular	18 (42.9%)
**Side of brain metastases**	
Left	13 (31.0%)
Right	12 (28.6%)
Both	17 (40.5%)
Number of lesions (median, range)	1 (1–8)
**Type of sistemic therapy**	
None	7 (16.7%)
Biological	10 (23.8%)
Chemotherapy	14 (33.3%)
Immunotherapy	4 (9.5%)
Chemotherapy + biological	7 (16.7%)
**Brain metastases surgery**	18 (42.9%)
**Radiotherapy for brain metastases**	37 (88.1%)
Whole brain	20 (54.1%)
Intensity modulated	0
Conformational	1 (2.7%)
Stereotactic	10 (27.0%)
Radiosurgery	6 (16.2%)
**Mutation/genic expression**	
None	27 (64.2%)
Egfr	4 (9.5%)
Her2	6 (14.3%)
Ckit	1 (2.4%)
Braf	2 (4.8%)
Alk	2 (4.8%)

SD, standard deviation; IQR, interquartile range; EGFR, estimated glomerular filtration rate; HER2, human epidermal growth factor receptor 2; CKIT, tyrosine protein kinase inhibitors; BRAF, murine sarcoma viral oncogene homolog B1; and ALK, anaplastic lymphoma kinase.

18 males and 24 females (57.1%), the mean age at the first visit in the epilepsy center was 52 years (±12 SD) and the median was 52, IQR = 45–62. The most frequent cancers were lung cancer (20 patients, 47.6%) and breast cancer (13 patients, 31%). The mutation/genic expression were: none in 27 patients (64.2%), EGFR+ 4 patients (9.5%), HER2+ 6 patients (14.3%), BRAF+ 2 patients (4.8%), ALK+ 1 patients (2.4%) and CKIT+4 1 patient (2.4%). The most frequent sites of brain metastases were: multilobular (18 patients, 42.9%), frontal lobe (9 patients, 21.4%) and parietal lobe (7 patients, 16.7%). In 17 patients (40.5%), the metastases were located on both sides. The types of oncological therapy at the time of the brain metastasis diagnosis were: chemotherapy (15 patients, 35.8%), biologic therapy (11 patients, 26.1%), chemotherapy+biological therapy (5 patients, 11.9%) and immunotherapy (4 patients, 9.6%). Seven patients were not undergoing any type of oncological therapy (16.6%). Median time between oncological diagnosis and first seizure: 19 months, ranging from –8.1 to 181.6 months (IQR = 2–68). Median time between brain metastasis diagnosis and first seizure: 0 months, ranging from –13.2 to 73.0 months (IQR = 0–6). For 22 patients (52.3%), the first seizure was at the same time of the brain metastasis diagnosis, for 4 patients it was before and for 16 (38.1%) it was after. In 25 patients, seizures were focal aware (59.5%), in 8 focal with impaired awareness (19.0%), in 5 focal to bilateral (11.9%), and in 4 (apparently) generalized (9.5%).

Among the 42 patients, 32 patients (76.2%) were given an ASM at the same time as the first seizure, one patient one month before, 4 patients within the month following the first seizure and 5 patients thereafter (after 2, 4, 6, 8 months). In 26 patients, the first ASM was LEV (61.9%; mean dose 1634 mg/day), followed by OXC (4 patients, 9.5%; mean dose 900 mg/day), PB (4 patients, 9.5%; mean dose 100 mg/day), VPA (3 patients, 7.1%; mean dose 1000 mg/day), LCM (2 patients, 4.8%; mean dose 200 mg/day), TPM (2 patients, 4.8%; mean dose 100 mg/day), and ZNS (1 patient, 2.4%; mean dose 200 mg/day). Right up to the last neurological control throughout the follow-up, 19 patients (45.2%) never changed the first ASM and maintained the same dosage: 12 with LEV at medium dosage 1375 mg/day, 2 OXC medium dosage 900 mg/day, 2 TPM dosage medium 100 mg/day, 2 LCM medium dosage 200 mg/day, 1 ZNS medium dosage 200 mg/day. All these patients remained seizure free until the end of the follow-up.

After a median period of seven months (range 1–145, IQR = 2.8–12.2), 16 patients (38.1%) required a change in therapeutic treatment due to seizures, and four (9.5%) maintained the drug but increased LEV dosage. Two patients changed therapy due to AEs (one for agitation with LEV at 1500 mg/day and one for psychiatric disorders with VPA at 1000 mg/day). Only one patient modified therapy due to interactions with systemic treatment (PB 100 mg replaced with LEV 2000 during therapy with AntiHer2). At the last follow-up available, 24 patients (57.1%) were seizure free (median follow-up of 9.1 months; IQR 3.1–16.7). Among these, 21 patients never changed their ASM monotherapy from the start (14 with LEV, 2 OXC, 2 TPM, 2 LCM, and 1 ZNS), while three had no seizures after the first change due to: AE in 2 patients (1 with LEV at 1500 mg/day due to agitation, changed with OXC 900 mg/day and one with VPA at 1000 mg/day owing to psychiatric disorders, changed with LEV 1000 mg/day), and in one patient who made an add-on (went from LEV 2500 to LEV 3000 mg/day plus LCM 200 mg/day). On the other hand, 18 patients (42.8%) never achieved freedom from seizures despite having made at least two therapy changes (median follow-up of 22.1 months; IQR 10.3–53.0).

We did not observe any significant correlation between seizure type (focal aware, focal with impaired awareness, and generalized seizures) and gender (*p* = 0.75), BM site (*p* = 0.99) or tumor histology (*p* = 0.90), and age (<65 or >65 years) (*p* = 0.76) (21).

**Figure 1:**
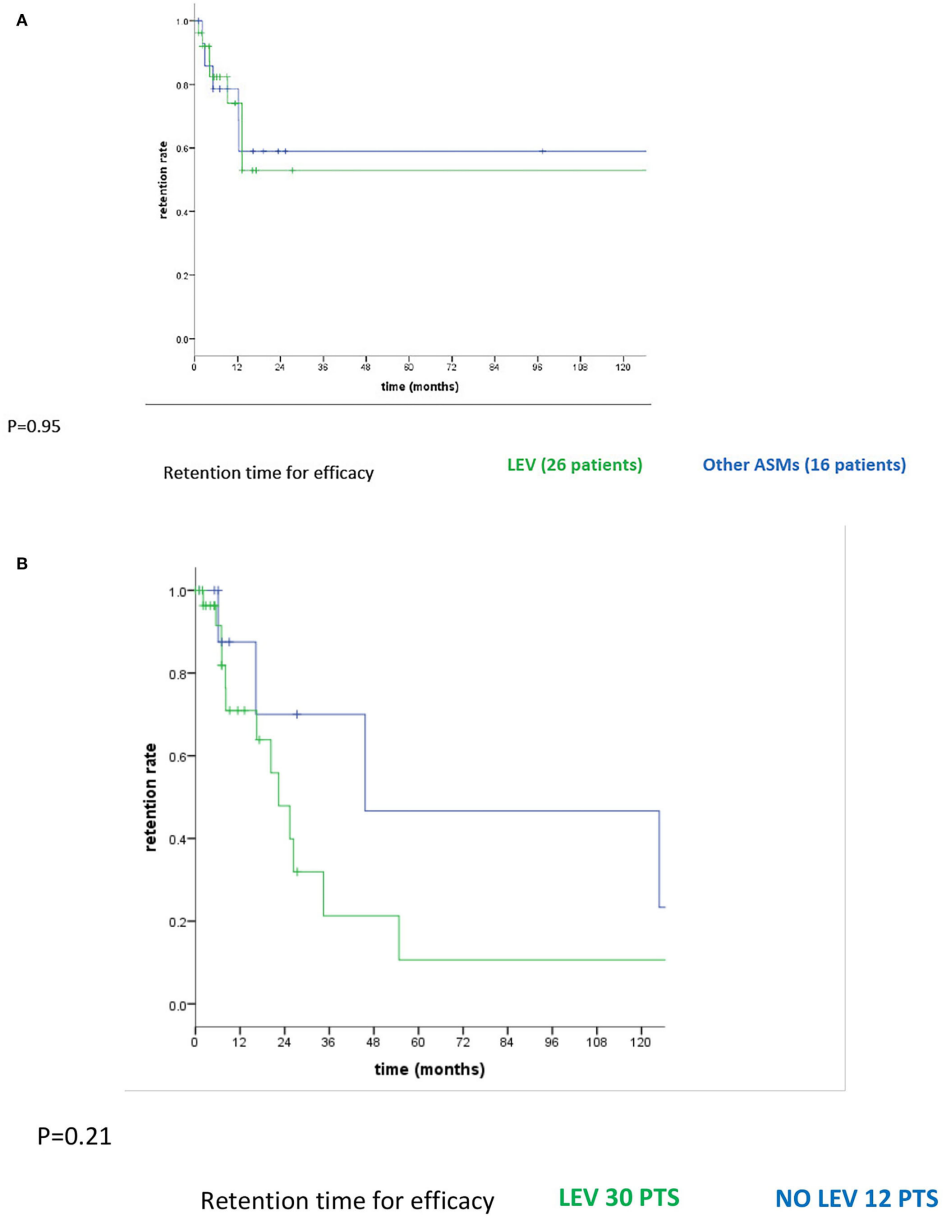
**(A)** Cumulative time-dependent probability of remaining on the first assigned ASM for the lack of efficacy between LEV and other ASMs. **(B)** Cumulative time-dependent probability of remaining on assigned ASM therapy for the lack of efficacy between LEV-based therapy and other ASMs after the first change due to inefficacy.

In the whole population (42 patients), the median follow-up time from the first seizure to the last follow-up was 12.4 months (range 0–161, IQR = 5.1–27.3). The median follow-up time from the first ASMs to the last follow-up was 11.4 months (range 0–161, IQR = 5.1–23.3). The cumulative time-dependent probability of remaining on the first assigned ASM for lack of efficacy did not vary between LEV and other ASMs (*p* = 0.95; Figure 1A). Even after the first change due to inefficacy, we did not find significant differences between LEV-based therapy (LEV monotherapy and LEV in polytherapy with other ASMs; 30 patients) and other ASMs (12 patients) (*p* = 0.21; Figure 1B) in the cumulative time-dependent probability of remaining on assigned ASM therapy for lack of efficacy. In order to evaluate if efficacy could differ between new and old generations of ASMs as the first monotherapy, we divided patients into three different groups: group 1 with patients with old generations of ASMs (PB in four patients and VPA in three), group 2 with new generations of ASMs excluded LEV (OXC in four patients, LCM in two, ZNS in one, and TPM in two patients), and group 3 with LEV (26 patients). Five out of seven patients in group 1 changed ASM due to inefficacy (71.4%); no ASM changes were made in group 2 (0%), while eight out of 26 patients in the LEV group changed for inefficacy (30.8%). We observed a significantly lower number of ASM changes due to inefficacy in group 2 compared with group 1 (*p* = 0.003). We did not observe any statistically significant differences in the number of ASM changes due to inefficacy in groups 2 and 3 (*p* = 0.062).

We also investigated whether the efficacy of ASM could be influenced by demographic or oncological variables, comparing the 13 patients who changed ASM monotherapy due to inefficacy (median time: 13 months) with the 29 patients who never changed (because seizure free) or changed ASM for other reasons, based on the abovementioned variables. We did not observe any significant differences regarding sex (*p* = 0.70), histology (*p* = 0.98), brain tumor site (*p* = 0.07), type of CT during epileptological follow-up (*p* = 0.36), time between diagnosis of brain metastases and first seizures (*p* = 0.78); all patients who changed due to inefficacy were younger than 65 years (*p* = 0.035) (21).

Furthermore we evaluated whether the efficacy of ASMs could significantly be influenced by oncological treatment underwent by patients; we did not observe any significant difference in the efficacy of ASMs based on the presence or absence of surgery (*p* = 0.10), different systemic therapies (none, chemotherapy, biological therapy, chemotherapy + biological therapy, immunotherapy; *p* = 0.30), and different radiotherapies (none, whole brain, stereotactic, radiosurgery; *p* = 0.13).

Regarding the DDD (16, 22, 23), in 19 patients the mean dosages of first ASM were 1375 mg/day (91.7% of DDD) for LEV, 900 mg/day (90% of DDD) for OXC, 200 mg/day (67% of DDD) for LCM, 100 mg/day (100% of DDD) for TPM and ZNS, and in 23 patients were 1857 mg/day (125% of DDD) for LEV, 900 mg/day (90%of DDD) for OXC, 1000 mg/day (66.6% of DDD) for VPA, and 100 mg/day (100% of DDD) for PB.

## Discussion

BMs are the most common cause of malignant central nervous system tumors 281 with up to 30–40% of cancer patients developing a metastasis at some point during the course of the disease (24). In these patients, the incidence of epilepsy as a consequence of BM remains as high as 15% to 25% (4). Data in literature reports that focal or generalized seizures are presenting symptoms in 15 to 20% of patients with BM, and are more common in patients with multiple metastases (6, 25–28) while a similar proportion of patients can be expected to develop seizures following diagnosis (6). In our study, among the 111 patients who had all variables required by the study at first visit, 95 (85.2%) had seizures and 16 did not (14.4%). This result it is very different to what the data in literature showed, that is approximately 14.6% of patients with BM experience seizures (4). This is probably due to the fact that, because our center is a specialized center certified in epilepsy by the LICE (Italian League against Epilepsy), almost all patients acceded either with a suspicion of epileptic seizures for which they needed diagnostic confirmation, or with a diagnosis of uncontrolled epilepsy for which a change on therapeutic treatment was necessary. Therefore, due to the characteristic of our center itself, we do not follow patients with non-epileptogenic metastases, which could be considered a selection bias.

On the other hand, it is possible that the percentage described in the literature could probably be underestimated. Indeed, data on the non-oncological epileptic population found in literature indicate how seizures may be unrecognized due to subtle semiology or may be misdiagnosed as syncope, migraine, or transient ischemic attacks: only by including an expert epileptologist early in patient screening could significant differences result in diagnostic accuracy (29, 30). Especially in cancer patients, a team evaluation performed by an oncologist and a neurologist together would reveal cases that are not recognized or underestimated.

Regarding our sample size it must be considered that it decreased from 111 to 42 patients, limiting our results. We hypothesize it could be due to the fact that this is a very fragile oncological patient population with a low life expectancy, therefore handle oncological aspect is the primary endpoint, compared to seizure control. Consequently, patients underwent regular oncological visits, but often they do not have a second epileptological examination after the first, referring to other non-specialized centers close to their area of origin or to their country (31). Thus this aspect makes it difficult to have long and serial epileptological follow-up in this patients population; however we believe that a multidisciplinary team approach with oncologist and neurologist working together, with periodic meetings and periodic follow-up for patients, could guarantee both a global and accurate taking care and could avoid patients' dispersion.

Regarding the risk of seizure we sought to characterize whether it could be influenced by oncological or demographical variables. Recent findings indicate that tumor type and location are the most important factors associated with the risk of seizures in brain metastases (32, 33). Among the most frequent tumor types, the highest rate of seizures is reported in melanoma, followed by lung cancer (33). In our population of 111 patients with all the variables required by the study at the first visit, we did not find significant differences between tumor type and the occurrence of seizures (lung vs. breast vs. melanoma vs. other, *p* = 0.62). We believe this result could be due to the characteristics of our center itself, as stated above.

Concerning the correlation between different brain regions and the risk of seizures, the risk is higher for patients with metastases involving or adjacent to brain regions with high epileptogenicity (motor cortex, temporal lobe, or multiple lesions) (33) or with parietal lobe lesions (34). Our results confirm this set of evidence, showing that the tumor location significantly affects the probability of having seizures also in our patients (all patients with parietal metastases, region with high epileptogenicity, have seizures; *p* = 0.012).

The relationship between demographic variables such as gender and age of patients and the occurrence of seizure has scarcely been studied, and the little data available are conflicting (34, 35). Witteler and colleagues (35) did not observe any significant correlation between age and seizure risk, while Raj Puri reported that age is the only variable that negatively correlates with the occurrence of pre and postoperative seizures (34). In our study, like Witteler and colleagues (35), we did not find any significant correlation between all these variables. All these studies are retrospective, and to better clarify this possible correlation, we believe that more prospective studies are necessary.

We found no significant correlation in 111 and 42 patients between gender, BM site, tumor histology, and seizure type. To our knowledge, there are no existing data in the literature reporting this aspect; we strongly believe that a prospective study may clarify this result.

Regarding the possible influence of demographic and oncological variables (histology, BM site, type of systemic therapy during follow-up, and time between diagnosis of BM and first seizure) on ASM efficacy, we observed a significant correlation only between ASM change due to inefficacy and patients' age (<65 years) (21). To our knowledge, no studies in the literature have explored this aspect. The only findings are by Beghi and colleagues (31). They recognize younger age (35 vs. 54 years) as a predictor of drug discontinuation due to lack of efficacy in brain tumor-related epilepsy patients. Thus, we cannot provide further explanation for this result; we believe that specific randomized controlled trials are needed to investigate this aspect in BM patients.

In our patient population at the last follow-up, 24/42 were seizure free (57.1%): 21 never changed their first ASM monotherapy and dosage, while three had no seizures after the first change. The remaining 18 patients (42.9%) never achieved freedom from seizures despite having had at least two changes in therapy. Our results confirm data in literature indicating that more than 50% of adults with tumor-related epilepsy respond to monotherapy, also in patients with BM (6, 36, 37).

Regarding the efficacy of different ASMs, 14 out of 24 patients were seizure-free in monotherapy with LEV (62.5%) and 1 with LEV plus LCM, while eight patients were in monotherapy with other ASMs. In patients with BTRE, different studies indicate that levetiracetam is a first-line option because of easy titration and few significant drug-to-drug interactions (3, 31, 37, 38), but also for its efficacy (3, 38). In fact, complete seizure control with LEV (as both add-on and monotherapy) in 47.4–88.9% of BTRE patients has been reported (37, 39–41). Our results also confirm the efficacy of LEV monotherapy in patients with epilepsy due to BM during systemic treatments.

Furthermore regarding the comparison between old and new generation ASMs our data seems to indicate that in our patients population old generation ASMs showed significantly less efficacy than the new ones, moreover, among the new generation ASMs both LEV and the other group (OXC, LCM, ZNS, TPM) seems to have the same efficacy. These data are in line with previous findings on brain tumor-related epilepsy patients, which report that LEV and/or new generations of ASMs are to be preferred over the old ones because they proved to have a scarce impact on hepatic metabolism (31). Considering that many oncological drugs have hepatic metabolism, but in particular immunotherapy, some biological molecules (e.g., cycline dependent kinase inhibitors 4/6, m-Thor inhibitors, PI3K inhibitors) and chemotherapy cause liver toxicity (42–44), the use of new generations of ASMs could reduce the occurrence of side effects caused by possible interactions with these oncological drugs (31, 42–44).

Constant monitoring of AEs during the follow-up of patients with BM and epilepsy must be considered of paramount importance, not only because AEs have a heavy impact on the quality of life of these patients (5), but also because decreased compliance due to AEs may lead to worse seizure control (1, 35, 45, 46). In our retrospective study, despite all patients undergoing oncological treatments during ASM therapy, only 2 of 42 changed ASM therapy for AEs (1 owing to agitation with LEV and one due to psychiatric disorders with VPA). The only two AEs we observed were neuropsychiatric, one with LEV and one with VPA, as already shown in the literature (38). Nevertheless, given the high efficacy, lack of drug interaction, and no need for drug-monitoring support, LEV remains a first-line agent (8).

Regarding the possible interactions between systemic therapy and ASM, we modified the ASM in only one patient in our population due to interactions with systemic treatment, withdrawing PB, an enzyme-inducer ASM, during therapy with AntiHer2. Probably because we mainly used new ASMs we had few AEs, and this is confirmed by the data in literature (8) indicating that in patients receiving chemotherapy, interactions with antitumor therapies should be considered possible when choosing the ASM and it is for these reasons newer generation drugs such as LEV should be preferred as first choice (8), as stated above.

Regarding Defined daily dose (DDD) (16) literature data reports that in non-cancer epileptic patients clinicians usually prescribe an antiepileptic dose lower than DDD, named Prescribed daily dose (PDD). Studies by Brodie et al. (22) and Horváth et al. (23) shown that in non-oncological epileptic patients, reach 75% of DDD is enough to obtian an effective seizure control. To the best of our knowledge there are no data relating to this topic in patients with BM. Therefore, we wanted to observe how the PDDs differed from the DDD regarding seizure control in BM patients who assumed the first monotherapy. Among our 42 patients, the mean daily dose of first ASM in the 19 patients who never changed their first ASM monotherapy and in the 23 patients who made at least one ASM change for any reason was very close to the DDD (see Supplementary materials). Considering our results, we believe that, unlike non-oncological epileptic patients, in patients with BM undergoing systemic treatments, it is necessary to maintain a similar DDD before considering an ASM ineffective in seizure control.

Furthermore, dosages close to DDD did not lead to an increase in AEs, despite the fact that our patients were all undergoing cancer treatment. To our knowledge, this is the first time that it is possible to correlate the DDD of an ASM in patients with brain metastases to efficacy. Future prospective studies with a larger population and a longer follow-up are needed.

## Conclusions

Our results suggest that the incidence of seizures in BM patients could be underestimated; only a close collaboration between oncologists and neurologists could contribute to identifying the incidence of seizures earlier and more accurately. Finally, in patients with BM and epilepsy undergoing systemic treatments, the choice of new ASMs is of paramount importance, both for the few AEs and for efficacy, but it is necessary to get close to DDD before considering an ASM ineffective in seizure control.

## Data availability statement

The datasets presented in this study can be found in online repositories. The name of the repository and accession number can be found below: GARRbox, https://gbox.garr.it/garrbox/index.php/s/PioGefMrE0WKVIu.

## Ethics statement

The studies involving human participants were reviewed and approved by Regina Elena National Cancer Institute IRCCS Ethics Commitee. Written informed consent for participation was not required for this study in accordance with the national legislation and the institutional requirements.

## Author contributions

MM: research hypotheses, data collection, interpretation of results, and manuscript preparation. AF: data collection, interpretation of results, and manuscript review. AM: data collection and manuscript preparation. EG and TK: data collection. DG: data analysis. All authors contributed to the article and approved the submitted version.

## Funding

This work was supported by Funds Ricerca Corrente 2022 from the Italian Ministry of Health.

## Conflict of interest

The authors declare that the research was conducted in the absence of any commercial or financial relationships that could be construed as a potential conflict of interest.

## Publisher's note

All claims expressed in this article are solely those of the authors and do not necessarily represent those of their affiliated organizations, or those of the publisher, the editors and the reviewers. Any product that may be evaluated in this article, or claim that may be made by its manufacturer, is not guaranteed or endorsed by the publisher.

## Abbreviations

BM: brain metastasis
ASMs: antiseizure medications
AEs: adverse events
ILAE: international league against epilepsy
LICE: italian league against epilepsy
CBZ: carbamazepine
CLZ: clobazam
PHT: phenytoin
PB: phenobarbital
VPA: valproic acid
TGB: Tiagabine
LTG: lamotrigine
LEV: levetiracetam
OXC: oxcarbazepine
PGB: pregabalin
TPM: topiramate
ZNS: zonisamide
LCM: Lacosamide
PER: perampanel
KPS: karnofsky performance status
DDD: defined daily dose
WHO: world health organization
SD: standard deviation
IQR: interquartile range
HER2: human epidermal growth factor receptor 2
BRAF: murine sarcoma viral oncogene homolog b1
EGFR: estimated glomerular filtration rate
CKIT: tyrosine protein kinase inhibitors
ALK: anaplastic lymphoma kinase
PDL1: programmed death ligand
PD1: programmed death.
