# Supplementary material for: Impact of epilepsy and its treatment on brain metastasis from solid tumors: A retrospective study
Source: Front Neurol. 2022 Oct 21;13:967946. doi: 10.3389/fneur.2022.967946 (PMC9634121; doi:10.3389/fneur.2022.967946)
Supplement: Supplementary file 1 [file Table_1.docx]

| **ASMs** | **DDD/mg** | **ASM’s mean dosage (mg) in patients who never change 1st ASM** |
| --- | --- | --- |
| LEV | 1500 | 1365 (n=12) |
| ZNS | 200 | 200 (n=1) |
| OXC | 1000 | 900 (n=2) |
| TPM | 300 | 100 (n=2) |
| LCM | N.A. | 200 (n=2) |
|  |  |  |

**Comparison between World Health Organization’s (WHO)^1^ ASMs Defined Daily Dose (DDD) and first ASMs’ mean dosages in the 19 patients seizure free who never change their first ASM monotherapy.**

**Comparison between World Health Organization’s (WHO) ASMs’ Defined Daily Dose (DDD) and ASMs’ mean dosages in the 23 patients who made at least one ASM change for any reasons.**

| **ASMs** | **DDD/mg** | **ASM’s mean dosage (mg) in patients with at least 1 previous ASM change** |
| --- | --- | --- |
| LEV | 1500 | 1857 (n=14) |
| PB | 100 | 100 (n=4) |
| VPA | 1500 | 1000 (n=3) |
| OXC | 1000 | 900 (n=2) |
|  |  |  |
|  |  |  |

**Legend:**

ASMs: Anti-seizure medications; DDD: Defined Daily Dose; LEV: Levetiracetam; ZNS: Zonisamide; OXC: Oxcarbazepine; TPM: Topiramate; LCM: lACOSAMIDE; pb: Phenobarbital; VPA: Valproic Acid.

^1^ Reference: WHO Collaborating Centre for Drug Statistics Methodology (WHOCC): DDD Definition and general considerations.
